# Supplementary material for: A Bayesian modelling framework for estimating tick-borne pathogen transmission dynamics at the host-tick interface
Source: PLoS Comput Biol. 2026 Apr 3;22(4):e1014146. doi: 10.1371/journal.pcbi.1014146 (PMC13068342; doi:10.1371/journal.pcbi.1014146)
Supplement: S1 Text — Classification of tick life stage and engorgement level. (DOCX) [file pcbi.1014146.s001.docx]

S1 Text for

**A Bayesian Modelling Framework for Estimating Tick-Borne Pathogen Transmission Dynamics at the Host-Tick Interface**

Younjung Kim^1,2*^, Bruno Faivre^3^, Thierry Boulinier^4^, Célia Sineau^3^, Clémence Galon^5^, Sara Moutailler^5^, Laure Bournez^6^, Raphaëlle Métras^1^

1 Sorbonne Université, INSERM, Institut Pierre Louis d’Épidémiologie et de Santé Publique (IPLESP), Paris, France

2 Current address: Department of Statistics, University of Oxford, Oxford, United Kingdom

3 Biogéosciences, UMR 6282 CNRS, Université Bourgogne-Europe, Dijon, France

4 CEFE, UMR 5175, CNRS, University of Montpellier, EPHE, IRD, Montpellier, France

5 ANSES, INRAE, Ecole Nationale Vétérinaire d’Alfort, UMR BIPAR, Laboratoire de Santé Animale, Maisons-Alfort, France

6 ANSES, Nancy Laboratory for Rabies and Wildlife, Malzéville, France

[younjung.kim@stats.ox.ac.uk](mailto:younjung.kim@stats.ox.ac.uk)

**Contents**

Figure A

Figure B

Figure C

Figure D

Classification of tick life stage and engorgement level

Reference


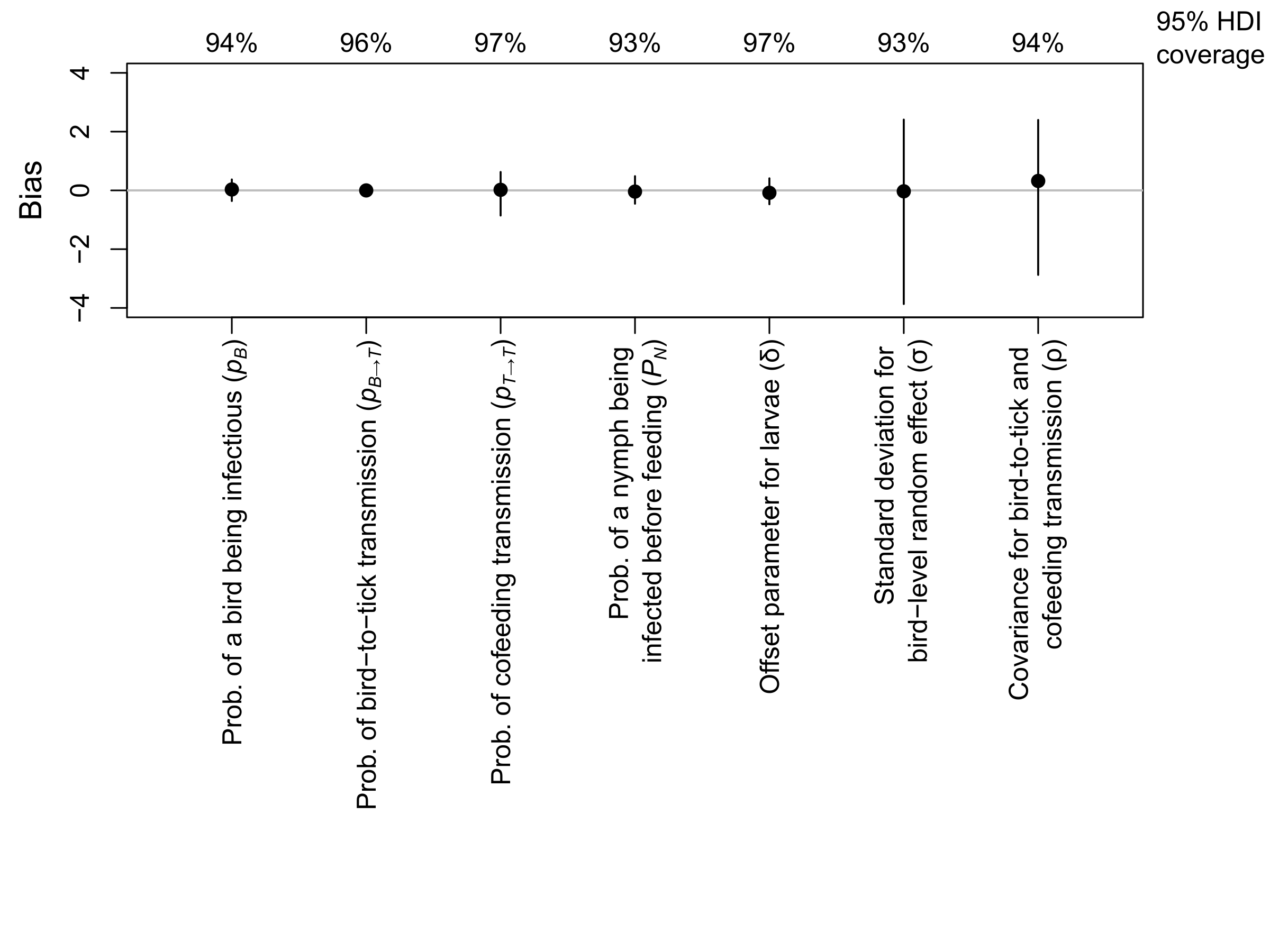


**Figure A.** Simulation-based assessment of the model’s ability to recover true parameter values. The x-axis represents the parameters estimated by the model, while the y-axis shows the absolute differences between the posterior median estimate and the parameter values used to generate the synthetic datasets to which the model was fitted. Circles indicate the medians, and vertical lines represent the 95% percentile intervals across 100 synthetic data fits. That is, for a given parameter, a bias of 0 indicates that its posterior median estimate is identical to the parameter value underlying the synthetic datasets. Percentage values above the graph show the percentages of the 100 synthetic data fits in which the parameter values underlying the synthetic datasets fall within the 95% highest density intervals [95%HDI] of the posterior estimates. See Table 1 and the Method section in the main text for the definition of parameters on the x-axis.


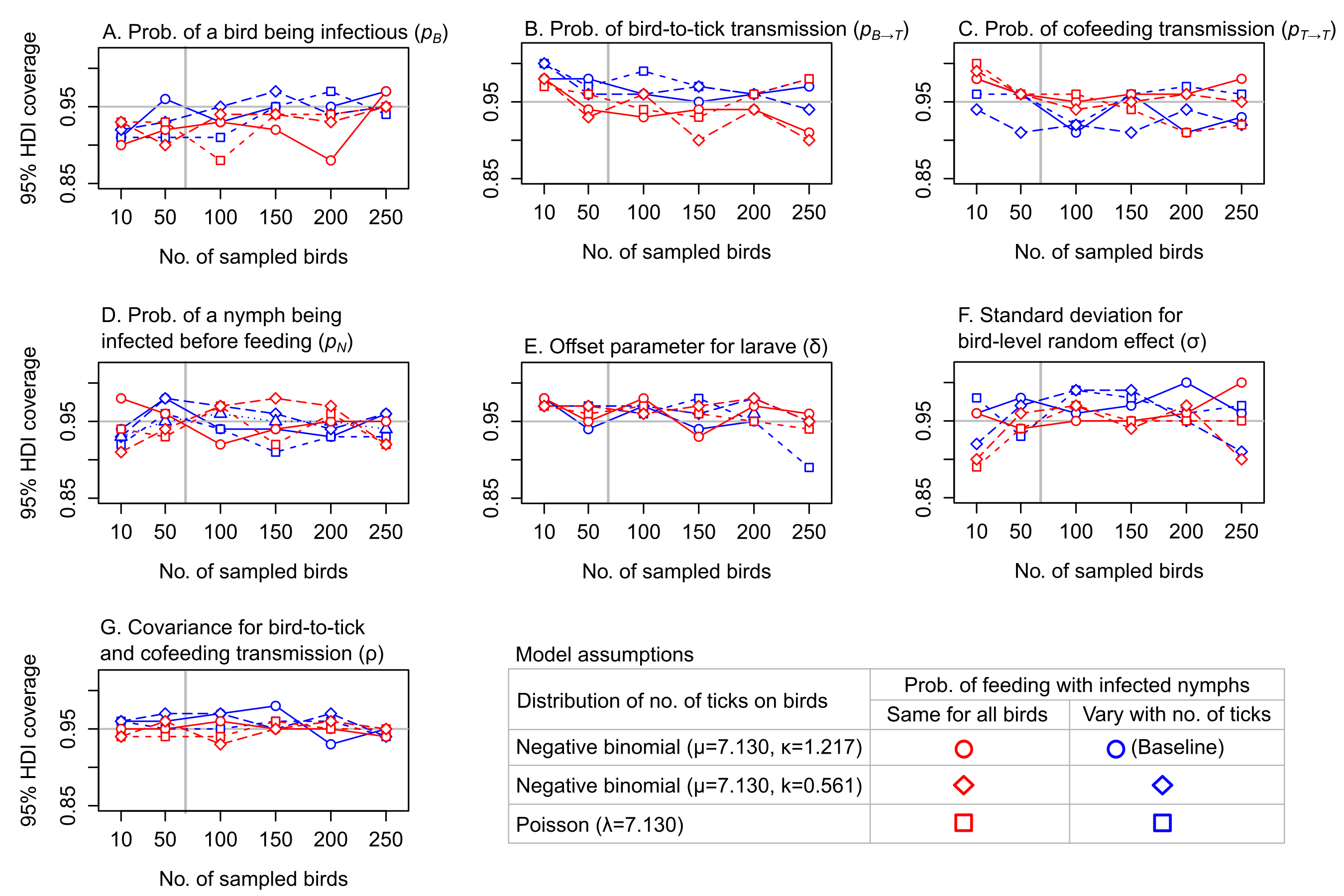


**Figure B.** 95%HDI coverage of posterior parameter estimates across different sample sizes, defined as the number of birds sampled (on the x-axis), and model assumptions (see the table in the figure). Point shapes indicate 95%HDI coverage under assumptions about the distribution of tick numbers on birds (overdispersed, Poisson, or constant), while point colours indicate 95%HDI coverage under assumptions about how the probability of cofeeding with infected nymphs varies (i.e. same for all birds regardless of the observed number of ticks on birds, or varying with the observed number of ticks on birds). Blue circles represent 95%HDI coverage under the baseline assumptions. The grey vertical lines indicate the sample size used in our empirical dataset.

**
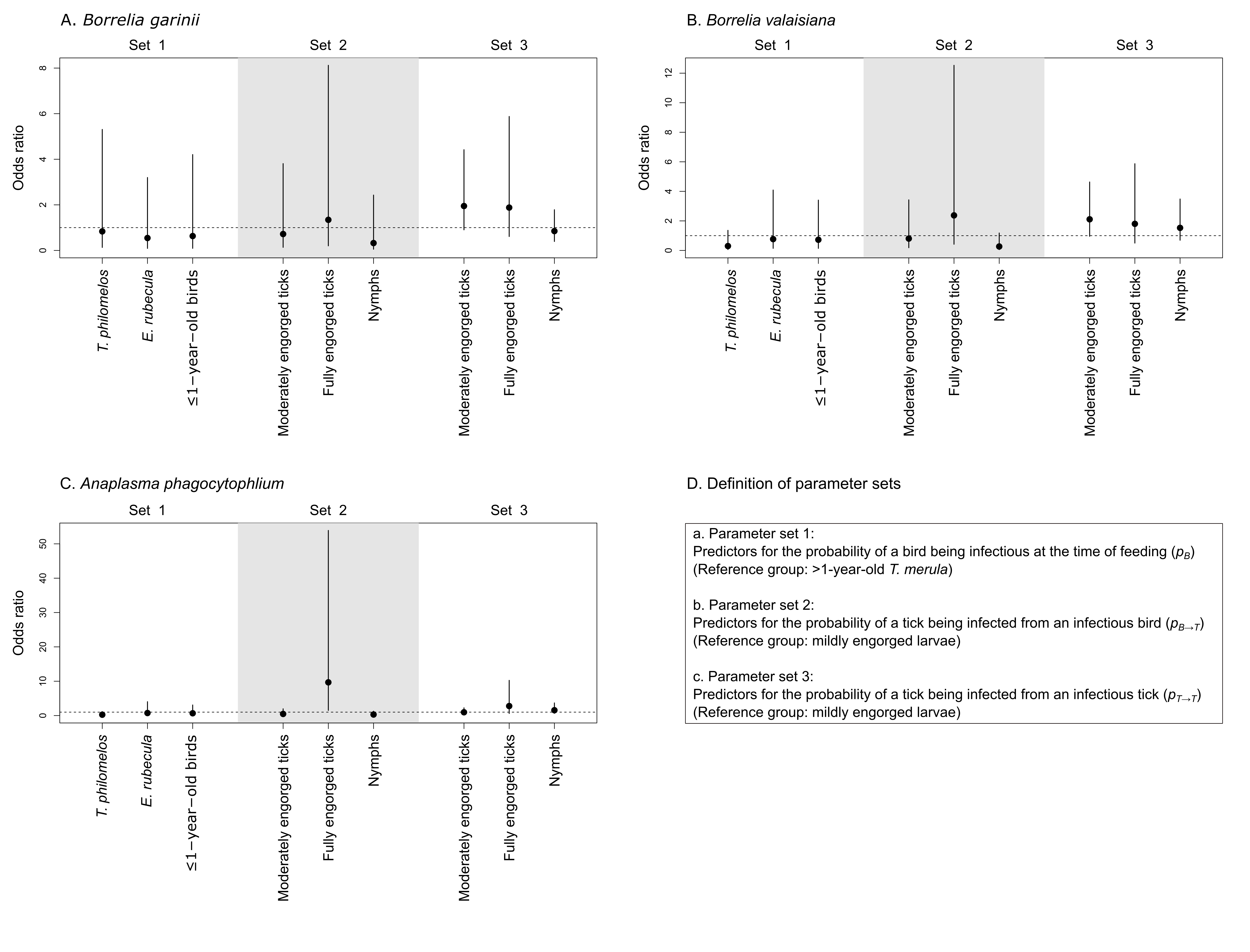
**

**Figure C.** Posterior parameter estimates from the full model. The results are expressed in odds ratios and presented by pathogen (panels A, B, and C). Circles indicate the median odds ratio estimates, and vertical lines represent their 95% highest density intervals, organized by parameter sets as defined in panel D.

**
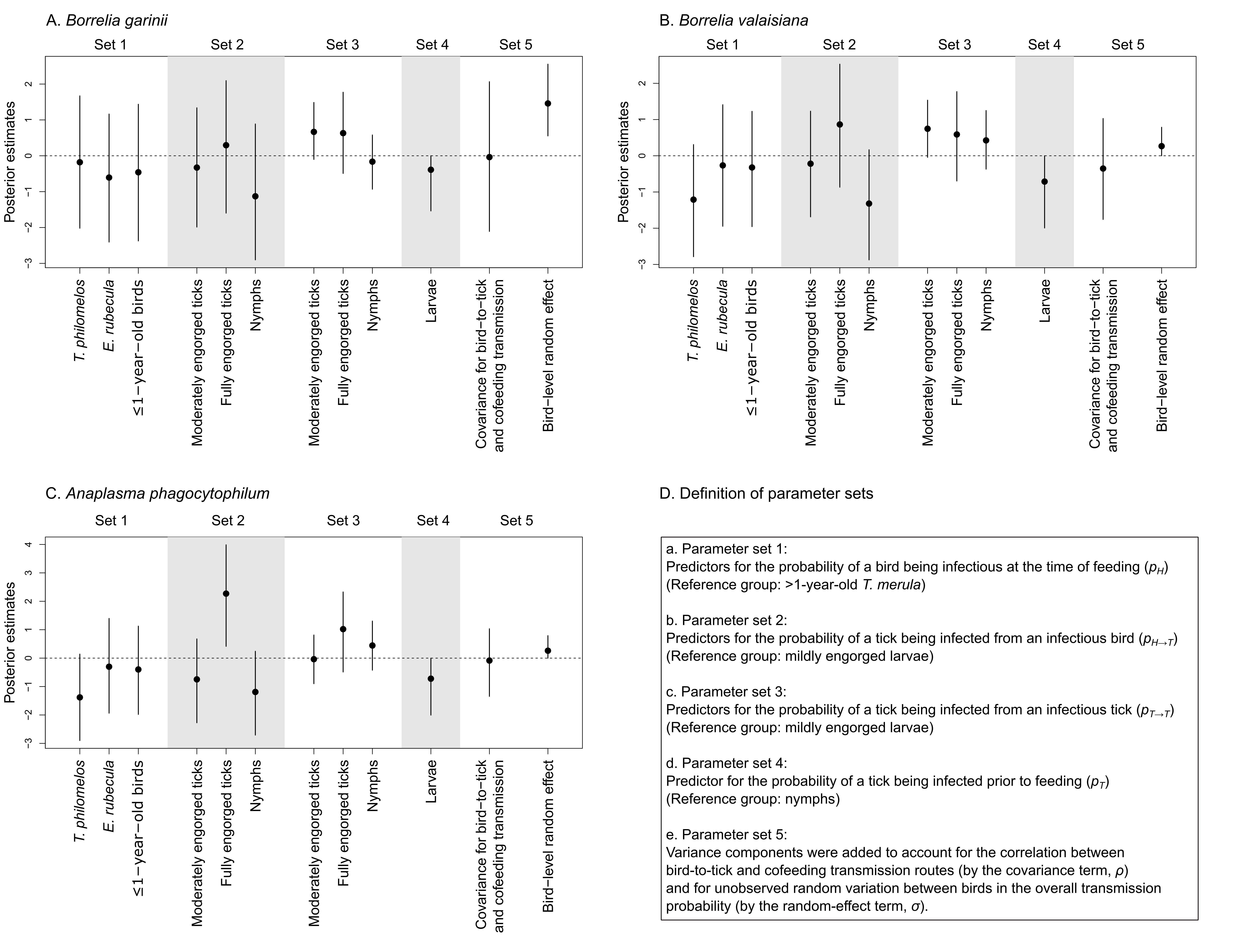
**

**Figure D.** Posterior parameter estimates from the full model. The results are presented by pathogen (panels A, B, and C). Circles indicate the median estimates, and vertical lines represent their 95% highest density intervals, organized by parameter sets as defined in panel D.

**Classification of tick life stage and engorgement level**

The life stage of ticks was identified morphologically using morphological keys (Pérez-Eid, 2007; Estrada-Peña et al., 2017). Engorgement levels were classified into three categories based on visual morphological changes with the following criteria:

- Mild: the tick is not engorged or only slightly engorged, with a narrow or slightly swollen body, and the scutum covering approximately half or slightly less than half of the dorsal surface
- Moderate: the tick is moderately engorged, with the body not fully rounded and the scutum covering between one-half and one-third of the dorsal surface;
- Full: the tick is fully engorged, with body greatly expanded and rounded, and the scutum covering less than one-third of the dorsal surface.

**Reference**

1. Pérez-Eid C. Les tiques: identification, biologie, importance médicale et vétérinaire. Paris: Lavoisier, Technique & Documentation; 2007.
2. Estrada-Peña A, D’Amico G, Palomar AM, Dupraz M, Fonville M, Heylen D, Habela MA, Hornok S, Lempereur L, Madder M, et al. A comparative test of ixodid tick identification by a network of European researchers. Ticks Tick Borne Dis. 2017;8(4):540-546. doi:10.1016/j.ttbdis.2017.03.001.
