# Supplementary material for: A Bayesian modelling framework for estimating tick-borne pathogen transmission dynamics at the host-tick interface
Source: PLoS Comput Biol. 2026 Apr 3;22(4):e1014146. doi: 10.1371/journal.pcbi.1014146 (PMC13068342; doi:10.1371/journal.pcbi.1014146)
Supplement: Attachment — Submitted filename: Response to Reviewer Comments.docx [file pcbi.1014146.s004.docx]

**Response to Editor and Reviewer Comments**

**Additional Editor Comments:** The Authors are expected to address all the criticisms by all Reviewers. In particular, please provide clearer justification of the specific host-vector components, host group, pathogens that were investigated (Reviewers #1, #2 & #3), provide more discussion of the quantitative insights, including how the estimated parameters translate into real-world implications, clarify “overdispersion”, provide a clear definition of “tested positive” (Reviewers #1 & #2), clarify sample collection period (Reviewers #1 & #3), assess the sensitivity of the model results to population size/demography (Reviewers #2 & #3) provide more description around sample size, provide more presentation of the results around the dataset and results, such as prevalences by species/age class, clarify the role of transovarial transmission, (Reviewer #1), clarified or discussed the minimum data requirement for the framework (Reviewer #2), provide a clear model formulation and calibration strategy and other methodological details (e.g. likelihood, free parameters, initial conditions, MCMC) (Reviewer #3).

**Response:** We appreciate the Editor for providing an opportunity to revise our manuscript based on the reviewers’ constructive and positive comments. We have addressed each comment point-by-point below, with particular focus on those highlighted by the Editor.

**Reviewers' comments**

Reviewer #1

**Comment 1.1**

General comments: The study develops an original framework that successfully isolates different transmission pathways using ecological and epidemiological field data. By combining simulations with applications to bird-tick datasets for three pathogens, the authors demonstrate that their approach reliably retrieves key parameters. The work further shows that incorporating fine-scale tick interaction data greatly strengthens inference. Overall, the framework represents a valuable and innovative contribution with clear practical relevance for designing future field studies.

Introduction: The introduction would benefit from clearer justification of the specific host-vector components investigated. The current text does not explain why the analysis focuses on bird-to-tick or tick-to-bird transmission, nor why birds were chosen as the focal host group in the first place. Similarly, the rationale for selecting the three pathogens, Ixodes ricinus, and the three bird species is not sufficiently developed. Providing broader context on Lyme disease ecology, particularly the roles of vertebrate hosts, the contribution of bird hosts, and the diversity of tick species and associated pathogens, would help readers understand the relevance of these choices. In addition, the discussion of sample size is ambiguous: the introduction does not specify whether sample size refers to numbers of hosts, ticks, or both, and clarifying this point early on would improve the reader’s understanding of the study’s objectives.

Materials and Methods: The Materials and Methods section is generally clear, well structured, and technically well argued. However, some important aspects remain implicit or insufficiently explained. For instance, it is unclear whether the model accounts for transovarial transmission, and (again) the examination of sample size effects is not explicitly defined. In addition, the dataset description lacks basic information such as the period of collection, which would help contextualize the analyses. Overall, the methodological framework would be strengthened by anchoring it more firmly in biological assumptions, for example by explicitly stating hypotheses related to sample size or overdispersion. This would improve interpretability and help readers connect the modelling choices to underlying biological processes.

Results: The Results section would benefit from presenting key empirical patterns from the dataset. In particular, reporting observed pathogen prevalence by bird species and age class, as well as prevalence in questing ticks and across tick developmental stages, would provide valuable context and help readers assess how the model outputs relate to the underlying data.

Discussion: The Discussion would benefit from addressing how the choice of bird species, the geographic origin of the field data, and the (unreported) sampling period may influence the results and their generality. More importantly, the section lacks a broader interpretation of the quantitative insights that the proposed framework can provide, particularly regarding the contribution of birds to the circulation of the studied pathogens. Placing the findings in this ecological and epidemiological context would substantially strengthen the discussion.

**Response:** We appreciate the reviewer’s positive and constructive comments the acknowledgment of our work’s contribution to the field. In the revised manuscript, we provide a clear justification for the selection of the tick-borne pathogens, bird species, and tick species studied. We also provide broader epidemiological context of Lyme disease and human granulocytic anaplasmosis in Europe. The sample size is clarified throughout the revised manuscript. The data are now described in detail, including a new table reporting the distribution of samples by bird- or tick-level variables and the proportion of each pathogen detected in the sampled ticks. We also explain the minimum data requirements for applying our framework. Finally, we provide additional information on the data including the sampling period and discuss potential applications of our framework to other host-tick systems are discussed. Please see below our point-by-point responses below. Line numbers are for the version with track changes.

Specific comments
Abstract

**Comment 1.2**

Line 26: The phrase “estimated key transmission parameters and associated factors” is too vague. Please specify which parameters and which associated factors are being estimated.

**Response:** We agree that the estimated key parameters and assessed factors were not clear in the abstract. The manuscript has been revised accordingly, specifying “probability of each transmission pathway” as estimated parameters and “bird species, tick life stage and engorgement level” as associated factors.

Lines 26 to 31

**Comment 1.3**

Line 29: The “impact of sample size” needs clarification. Does sample size refer to numbers of hosts, ticks, feeding events, or another unit?

**Response:** In our framework, inference is informed by both the number of hosts and the number of ticks sampled from these hosts and tested. However, in typical host-tick field designs where the number of ticks per host follows a specific distribution, the total number of ticks collected is largely determined by the number of hosts sampled. The abstract sentence has been removed during revision to address other reviewer comments. However, throughout the revised main text, we define “sample size” as the number of sampled hosts (birds in our case study), with further explanation provided in the Methods.

Lines 124, 313, 356 to 365

**Comment 1.4**

Lines 30-31: The statement “field data collected at the bird-tick interface” lacks essential contextual details. Please indicate where and when these data were collected.

**Response:** The bird-tick data were collected in Forêt Domaniale de Haye region in Northeast France in 2023. “Northeast France in 2023” is now stated in the abstract, and the forest name is stated in the main text.

Lines 128, 397

**Comment 1.5**

Line 35: The sentence about improved performance when more information on variability in cofeeding probabilities was available should be expanded. What are the practical implications of this finding for field data collection and future research? As this is a key result, readers need to understand its consequences.

**Response:** We thank the reviewer for highlighting this result as a key finding. Variability in cofeeding probabilities could be informed by collecting the spatial distribution of sampled ticks on the host and their infection status. In the discussion, we suggest that future research applying our framework should collect these data in greater detail. Following the reviewer’s suggestion, we have also revised the abstract to provide the implications for future studies.

Lines 38 to 42, 679 to 692, 713 to 727

**Comment 1.6**

Lines 39-40: The claim that “Cofeeding transmission was found as a significant factor for all three pathogens” is too imprecise. Please indicate for which outcome or parameter cofeeding transmission was significant.

**Response:** The sentence indicated a significantly better model fit for the model allowing cofeeding transmission compared to those not allowing it. The manuscript has been revised to clarify this.

Lines 44 to 47

Introduction

**Comment 1.7**

Line 63: The focus on “bird-to-tick or tick-to-bird” transmission is not sufficiently justified. Other hosts play important roles in the system, and the introduction should explain why the study focuses specifically on birds.

**Response:** The phrase “bird-to-tick or tick-to-bird” in the first sentence of the Introduction was misleading because our framework is applicable to any host-tick system, and we used the bird-tick system as a case study. We have therefore revised this to “host-to-tick or tick-to-host”. In the present study, we focused on the birds and their ticks as they are key actors in the transmission of the pathogens considered. *Borrelia garinii* and *B. valaisiana* show strong host associations with birds, while *Anaplasma phagocytophilum* is a contrasting bacterial pathogen commonly detected in birds with potentially different transmission ecology. Studying these pathogens within a bird-tick system thus enables a within-system comparison to evaluate whether our framework can resolve pathogen-specific dynamics. Additionally, we had external datasets from the same region with which to evaluate the reliability of the model estimates. The rationale for selecting the bird–tick system as a case study has now been added to the revised manuscript.

Lines 75, 128 to 150

**Comment 1.8**

Line 110: The phrase “assess the effect of sample size” needs clarification. Specify whether this refers to the number of hosts, ticks, feeding events, or another sampling unit.

**Response:** Please see our response to Comment 1.3 above

**Comment 1.9**

Lines 112-116: The introduction should better contextualize the selected hosts, tick species, and pathogens, and justify why these particular organisms were chosen. In particular, the relevance of including Borrelia valaisiana, which typically shows low prevalence, should be addressed.

**Response:** Please see our response to Comment 1.7 above

Materials and Methods

**Comment 1.10**

Lines 131-133: The sentence beginning “The underlying framework… small mammals” presents a forward-looking perspective. This type of content belongs in the Discussion section.

**Response:** The sentence has been removed.

Lines 165 to 167

**Comment 1.11**

Lines 245-246: The statement “Nymphs are assumed to have a higher probability than larvae” requires clarification regarding transovarial transmission. How is transovarial transmission from infected females to larvae taken into account in the model? Are larvae initialized with zero infection, or with a non-zero baseline prevalence? If so, what value is used, and does it vary by pathogen?

**Response:** To clarify, the model estimates the probability that a tick is already infected prior to feeding at its sampled life stage, separately for larvae and nymphs. However, we do not directly model the probability of transovarial transmission from an infected female adult to her offspring. We account for prior infection in both larvae and nymphs using separate parameters, and these parameters were estimated (please see panel D in Figures 3, 4, and 5). The method section describing the prior-infection probability has been expanded to clarify these points.

Lines 273 to 286

**Comment 1.12**

Line 253: The term “sample size” again needs to be specified.

**Response:** Please see our response to Comment 1.3 above

**Comment 1.13**

Line 255: The phrase “to assess the impact of overdispersion of the data (ticks are clustered by birds)” is too concise. Explain briefly how overdispersion is expected to influence parameter precision and why this is important for the system.

**Response:** We examined how heterogeneity in tick burdens across birds affects estimation performance, because it changes both the amount and distribution of information in the data and can therefore influence model fitting. The direction of such influence is hard to expect without analysis since it would depend on the frequency of transmission events, how cofeeding risk scales with tick burden, and how much unobserved between-bird variation exists. We have revised the manuscript to clarify these points.

Lines 315 to 317

**Comment 1.14**

Line 265: The text refers to “mean… empirical data” but does not provide the corresponding results. Please report these empirical values.

**Response:** The values have now been reported in the manuscript.

Lines 327 to 329

**Comment 1.15**

Line 268: Explain why only Borrelia garinii was used at this step.

**Response:** In the simulation-based model assessment, we adopted two different approaches for the probability of cofeeding with infected nymphs. The first approach assumed that this probability varies with the observed number of ticks on birds, whereas the second approach assumed that it remains constant across birds regardless tick infestation level. We used *Borrelia garinii* dataset for these simulations because it showed a significant association between the presence of at least one infected nymph and the observed number of ticks on individual birds. This association allowed us to implement and compare the two approaches (Corresponding to two columns in the model assumptions table in Figures 1 and 2). The justification for this choice is provided in a previous paragraph. In the revised manuscript, we further clarified the link between these paragraphs by naming the two approaches “Cofeeding probability assumption 1” and “Cofeeding probability assumption 2”.

Lines 339 to 345

**Comment 1.16**

Line 269: The expression “using randomly selected parameter values” is unclear. Specify how these parameters were chosen, from which ranges, and for what purpose.

**Response**: We have clarified the relevant sentences, with justifications for how parameter values were selected.

Lines 329 to 339

**Comment 1.17**

Paragraph 2: Impact of sample size: As previously noted, define which sample size is being examined. Also, articulate any biological or statistical hypotheses regarding how sample size is expected to affect the estimates.

**Response:** See our response to Comment 1.3 above for the definition of sample size. For the second request, the exact impact of sample size is difficult to predict because the complexity of the host-tick system. The numbers of hosts and ticks, as well as the level of infection in each, jointly affect parameter estimation. Of course, parameter estimation will be improved with increasing sample size, but it is challenging to make specific hypotheses about how sample size will affect individual parameters within the jointly estimated framework. Therefore, we avoided proposing a specific priori hypothesis about how sample size would affect parameter estimation. Instead, our assessment can be used in future research to develop hypotheses about sample size requirements before designing a sampling scheme. In particular, we highlight that our framework is based on a sampling scheme that reflects field practice, and could therefore be applied to inform sampling design in future research on host-tick systems.

**Comment 1.18**

Paragraph 3: Impact of tick overdispersion: Similarly, clarify the hypotheses being tested. What effects do you expect overdispersion (tick aggregation on hosts) to have on estimation accuracy?

**Response:** We examined how heterogeneity in tick burdens across birds affects estimation performance considering scenarios with varying degrees of overdispersion as well as models that do not assume overdispersion. However, as with sampling size, its impact depends on both the amount and distribution of information in the data, including the level of infection in hosts and ticks, how cofeeding risk scales with tick burden, and the extent of unobserved between-bird variation. Therefore, within the jointly estimated framework, we avoided proposing a specific a priori hypothesis about how overdispersion would affect parameter estimation. Instead, our assessment could inform future research on host-tick systems to make specific hypotheses.

**Comment 1.19**

Line 297: “for tick distributions” should be clarified: are these distributions on birds, on questing ticks, or both?

**Response:** It is the distribution of the number of ticks per bird. The part has now been clarified.

Lines 382 to 390

**Comment 1.20**

Line 304: Indicate the period of collection (months/season and year). Tick phenology and pathogen prevalence vary strongly with time.

**Response:** The required information is now included in the revised manuscript.

Lines 396 to 398

**Comment 1.21**

Line 310: Erithacus rubecula is no longer part of Turdidae. Also correct the spelling: remove the final “r” in rubecular.

**Response:** We thank the reviewer for finding out this typo and providing updated information on *Erithacus rubecula*. The manuscript has been revised accordingly.

Lines 400 to 404

**Comment 1.22**

Line 313: The statement “on the head and legs, where ticks are more found” should be supported with a reference. Head infestation is common, but high tick loads on legs are not commonly reported.

**Response:** We agree with the reviewer that ticks are most commonly found on the head. We have revised the sentence with a reference.

Lines 405

**Comment 1.23**

Line 317: Justify the decision to collect a maximum of only 10 ticks per bird. How might this choice affect prevalence estimates or parameter inference?

**Response:** We fully agree with the reviewer that not testing all identified ticks could have some impact on parameter estimation. In particular, engorged ticks were prioritised when there were more than 10 ticks on the sampled bird, to maximise the likelihood of pathogen detection. We expect that depending on the association between tick engorgement and pathogen positivity and the clustering of infected ticks among others, our sampling design could have biased pathogen prevalence and downstream transmission probability estimates. This is now discussed this in the revised manuscript.

Lines 408 to 412, 700 to 706, 713 to 727

**Comment 1.24**

Line 334: Again, specify the collection period. The timing strongly influences observed prevalence and should be reported.

**Response:** The manuscript has been revised accordingly.

Lines 430

Results

**Comment 1.25**

Figure 2.E: The title contains a typo: replace “larave” with “larvae”.

**Response:** We thank the reviewer for finding this mistake. The type has now been corrected.

**Comment 1.26**

Line 398: Replace “studies” with “studied.”

**Response:** We thank the reviewer for finding this mistake. The type has now been corrected.

Line 503

**Comment 1.27**

Line 436: The statement “with no significant difference observed between models that did or did not assume prior infection at the larval stage” requires more explanation. Please clarify: (i) how transovarial transmission was incorporated (or not) into the model, (ii) whether larvae were initialized with zero or non-zero infection levels, (iii) and what the literature reports regarding expected larval infection prevalence for each of the three pathogens studied.

**Response:** See our response to Comment 1.11 above. The probability that a larva is infected via transovarian transmission is modelled with Eq. 11, where $p_{s(i)}=p_{L}$. In the model that did not assume transovarian transmission, $p_{L}$ was not included in Eq. 11 and, therefore, ${FOI}_{3}\left[ i,k \right]={FOI}_{2}\left[ i,k \right]$. The Method and Result sections have been revised to clarify these differences.

In addition, $p_{L}$ is modelled with a non-informative prior; that is, no assumption was made about the distribution of this parameter. Finally, the estimated probability that a larva was infected via transovarian transmission was very low for the three pathogens, and assuming this probability did not significantly improve model fit from the model that did not assume it. This result is consistent with the literature, which supports no or rare detection of the pathogens in *Ixodes ricinus* larvae. This result has been discussed in the revised manuscript.

Lines 543 to 554, 671 to 677

**Comment 1.28**

Figure 5: The same symbol (red triangle) appears to represent different quantities in panels A and D, creating confusion. Please modify the symbols and legend to avoid ambiguity.

**Response:** We thank the reviewer for finding this mistake. The figure has been revised to remove the ambiguity.

**Comment 1.29**

Figure 5.A: Explain why observational data are shown only for Turdus merula and not for the two other bird species.

**Response:** PCR was performed only on samples collected from *Turdus merula*. Thus, there was no PCR test result for the other two bird species.

**Comment 1.30**

Figure 5 – legend: The phrase “proportion of T. merula birds that tested positive to A. phagocytophilum” is ambiguous. Does “tested positive” mean that at least one tick collected from a bird was infected? If so, please state this clearly; if not, specify the criterion used.

**Response:** The phrase indicates the proportion of *T. merula* birds whose blood samples tested positive to *A. phagocytophilum*.


Discussion

**Comment 1.31**

Line 498: The term “critical factor” is vague. Rather than using qualitative language, explain why cofeeding is important for transmission in this system. What does this result imply? For instance, does the model suggest that cofeeding contributes a substantial, and highly variable, proportion of overall transmission? Clarifying this would help readers understand the biological significance of the finding.

**Response:** A new sentence has been added to discuss its significance in the context of pathogen persistence.

Lines 644 to 650

**Comment 1.32**

Line 518: The statement that the framework “could provide more realistic estimates” should be nuanced. The empirical datasets used here are relatively small and restricted to a few bird species and only one site, which limits the generality of the conclusions. Acknowledge these constraints when discussing realism.

**Response:** We have deleted the sentence because it was somewhat repetitive of the preceding text. We have also added “in the studied host and tick populations” to clarify the scope of generalisability.

Lines 667

**Comment 1.33**

Lines 403-406: The explanation for this result is missing. Please expand on the biological or methodological mechanisms that could account for this pattern.

**Response:** In the original manuscript, this unique finding for *A. phagocytophilum* was discussed by comparison with the Borrelia species with respect to potential replication sites in the host. The significant association between tick engorgement level and the probability of bird-to-tick transmission may be explained by the fact that *A. phagocytophilum* replicates predominantly within blood neutrophil granulocytes. Thus, greater engorgement (reflecting longer feeding and a larger blood meal) could increase the likelihood of ingesting infected cells. In contrast, such an association may be less apparent for Borrelia species, as they disseminate and persist more extensively in tissues than *A. phagocytophilum*.

Lines 636 to 643

**Comment 1.34**

Lines 425-428: The interpretation of these results should be placed in the context of the field prevalence of the three pathogens. Without this perspective, it is difficult to assess how representative or meaningful the modelled effects are.

**Response:** Comparing co-feeding probabilities with the prevalence of the three pathogens in questing ticks would be challenging and not directly comparable. Questing ticks generally feed on a variety of hosts, and therefore the level of pathogen circulation observed at the bird–tick interface is likely to differ from prevalence in questing ticks. For such a comparison to be meaningful, dynamic models that integrate longitudinal data from questing ticks and multiple host–tick systems would be needed. Our model estimates could inform one component of such models.

References

**Comment 1.35**

Several references contain typographical errors or missing letters (e.g., urdus instead of Turdus, orrelia instead of Borrelia in the reference number 13). A thorough revision of the reference list is needed to correct these types of errors.

**Response:** We appreciate the reviewer for finding these errors. They are now corrected in the revised manuscript.

Reviewer #2

**Comment 2.1**

This manuscript presents a well-structured and scientifically compelling modelling framework for investigating the transmission dynamics of tick-borne pathogens at the host–tick interface. By integrating multiple infection pathways within a Bayesian inference approach informed by field data, the study provides a robust quantitative basis for identifying key drivers of pathogen transmission. The manuscript is clearly written, methodologically rigorous, and conceptually engaging. In a few sections, the narrative could be streamlined to enhance the practical applicability of the framework, and the inclusion of concrete examples could help readers better understand how the estimated parameters translate into real-world implications. Overall, the manuscript fits well within the scope of the journal and constitutes a valuable contribution to the field.

**Response:** We appreciate the reviewer’s constructive and positive comments as well as the acknowledgement of the importance of our work in the field. We have addressed the reviewer’s comments point-by-point below. Line numbers are for the version with track changes.

Minor comments

**Comment 2.2**

Line 34. I am not fully convinced by the use of the word “true”; “realistic” may be more appropriate in this context?

**Response:** By “true”, we mean the parameter values used to simulate host-tick pathogen data. We fitted the model to these simulated data to assess whether it could recover the underlying parameter values. We have clarified the sentence in the revised manuscript.

Lines 36 to 38

**Comment 2.3**

Lines 52–53 (and similarly line 68). It is very interesting that the framework is presented as applicable to routinely collected field data. However, I wonder whether host-based tick sampling can truly be considered routine. In many settings, routine surveillance more commonly involves collecting ticks from the environment, whereas host-derived ticks may be less frequently available and more challenging to obtain, particularly for certain wildlife species. I would suggest clarifying or slightly expanding this point to acknowledge these practical differences while still emphasising the value of the proposed approach.

**Response:** We fully agree with the reviewer. By “field studies”, we meant studies that sample hosts and their ticks. In such studies, the data used in our framework are routinely collected, although more completeness in data collection would improve estimation performance as discussed in the manuscript. Throughout the revised manuscript, we have explicitly stated that “field studies” refers to studies that sample hosts and their ticks. In addition, as the reviewer suggested, we have highlighted the value of such field studies, combined with our approach, while acknowledging the logistical and ecological challenges involved, particularly compared with most surveillance that focuses on questing ticks.

Lines 64, 86 to 90, 112 to 115

**Comment 2.4**

Line 54. The manuscript does not explain why authors chose these three systems instead of others. Providing a brief biological rationale would strengthen the argument and improve replicability.

**Response:** In the revised manuscript, we have expanded the justifications for selecting the tick species, tick-borne pathogens, and host species in the Introduction section.

Lines 128 to 150

**Comment 2.5**

Lines 76–77. I am not entirely sure that obtaining all the required information—especially age-structured sampling, for instance—is straightforward in wildlife studies or routine surveillance. A similar consideration applies to lines 127–128. Could author expand including this limitation or explaining why this is not a limitation for replicability of the study?

**Response:** We fully agree with the reviewer that it is difficult to precisely determine the age of individual hosts in field studies. This is why we used an age-group classification for birds (i.e. younger or older than one year). The impact of age (or other host variable)-stratified sampling is beyond the scope of this study.

Regarding the second comment, the mentioned sentence is about a bird-level random effect we included in the model to account for a bird-level variability that could not captured by the measured covariates. We do not consider inclusion of this random effect to be a limitation. Rather, it is an effective way to address unobserved heterogeneity among individual birds, and we do not expect to collect all the data required to fully characterise such variability in the field.

**Comment 2.6**

Line 103. It is unclear whether infection risk refers to the risk for birds, ticks, or both. Clarifying this would improve readability.

**Response:** It refers to the risk for ticks because the model considers host-to-tick transmission, cofeeding transmission, and infection prior to feeding. The manuscript has been revised accordingly.

Lines 116

**Comment 2.7**

Line 130. I would recommend expanding this section by briefly explaining the rationale for choosing this particular system. What features make it a suitable case study, and which characteristics support generalisation to other host–tick systems? Conversely, are there systems where this framework would not be applicable? For instance, line 133 mentions the possibility of extending it to small mammals, why specifically small mammals, and not mammals more broadly?

**Response:** In the revised manuscript, we have expanded the justifications for selecting the tick species, tick-borne pathogens, and host species in the Introduction section.

Regarding the second comment, the model can certainly be applied to hosts other than small mammals, but it would require model adaptations depending on the assumed roles of hosts. For Borrelia species, large mammals, for example, deer, would not act as an amplifying host. Therefore, it is logical not to consider a host-to-tick transmission pathway. We have mentioned this applicability briefly in the revised manuscript.

Lines 614 to 624

**Comment 2.8**

Line 162. It may be helpful to clearly state which bird-specific characteristics need to be considered in the modelling framework, to guide readers who may be less familiar with the system or this kind of modelling.

**Response:** The minimum requirements for the modelling framework are now given in the Method section. The bird- and tick-level characteristics considered in the study are now also provided in the main text and Table 1.

Lines 177 to 182 and Table 1

**Comment 2.9**

Line 245. It would be useful to clarify what is meant by higher in this context. Does it refer to the constraint that nymphal pTinf must just somehow exceed that of larvae, or does it imply something else?

**Response:** The model specification in the original manuscript was not clear. Yes, the probability of infection prior to feeding was assumed higher for nymphs than larvae, because nymphs have additional feeding at their larval stage. We have clarified this process in the revised manuscript.

Lines 289 to 300

**Comment 2.10**

Line 263. Is there any information available on the sensitivity of the model to population size? A brief comment on this would strengthen the interpretation of the results.

**Response:** The first simulation-based assessment, which included the sentence mentioned above, evaluated the model’s ability to recover underlying parameter values given the sample size of the bird-tick pathogen data. The impact of the population size, that is, the number of birds sampled (and, implicitly, the number of ticks collected from these birds), was assessed in the second simulation-based assessment.

**Comment 2.11**

Line 286. It is not entirely clear which model is being referred to here when you write “a model”. Providing a more explicit reference would improve clarity.

**Response:** We have clarified the sentence in the revised manuscript and also directed readers to the relevant sections.

Lines 366 to 371

**Comment 2.12**

Lines 288–290. Could you clarify why the system showing a significant association between the presence of at least one infected nymph and the estimated number of ticks was selected for this analysis?

**Response:** It is because it allows us to evaluate different assumptions about cofeeding probability, as part of the general assessment of the model structure. If there is no significant association in the underlying simulated data, it would not be possible to assess whether considering that association improves estimation performance (i.e. blue points in Figures 1 and 2). We have clarified this in the mentioned paragraph as well as in other relevant paragraphs.

Lines 339 to 345

**Comment 2.13**

Line 317. Could the authors expand on the rationale behind this choice, and behind selecting the value of 10?

**Response:** We fully agree with the reviewer that not testing all identified ticks could have some impact on parameter estimation. In particular, engorged ticks were prioritised when there were more than 10 ticks on the sampled bird, to maximise the likelihood of pathogen detection. We expect that depending on the association between tick engorgement and pathogen positivity and the clustering of infected ticks among others, our sampling design could have biased pathogen prevalence and downstream transmission probability estimates. This is now discussed this in the revised manuscript.

Lines 408 to 412, 700 to 706, 713 to 720

**Comment 2.14**

Line 330. Do the two methods yield completely identical results? If not, how comparable are they? Why did the authors chose this method? A brief comparison would strengthen this section.

**Response**: For each pathogen, the subset of the positive samples (around 20%) was tested also by conventional PCR to check that microfluidic-based testing results are reliable. We confirmed >99% sequence homology in all these samples. We have clarified this part in the revised manuscript. The detailed approach can be found in the cited references published by co-authors.

Lines 423 to 426

**Comment 2.15**

Line 332. Is there a way to (even roughly) standardise the tick classification used here? For applying this framework to other datasets, it would be helpful to know whether the same categories can be used and how they might be replicated.

**Response:** Tick life-stage and species were identified morphologically using morphological keys (Pérez-Eid, 2007; Estrada-Peña et al., 2017). Engorgement levels were classified into three categories based on visual morphological changes:

Mild: the tick is not engorged or only slightly engorged, with a narrow or slightly swollen body, and the scutum covering approximately half or slightly less than half of the dorsal surface

Moderate: the tick is moderately engorged, with the body not fully rounded and the scutum covering between one-half and one-third of the dorsal surface;

Full: the tick is fully engorged, with body greatly expanded and rounded, and the scutum covering less than one-third of the dorsal surface.

This information is now included in the revised Supplementary Materials.

**Comment 2.16**

Line 334. For the sake of reproducibility, it may be beneficial to add a brief description of how these data were collected.

**Response:** We have added more information on the mentioned part.

Lines 427 to 439

**Comment 2.17**

Line 368. The captions of Figures S1 and S2 could be improved. At present, the figures are not very clear, and the captions are not sufficiently self-explanatory. Providing a few additional details would help readers interpret the results more easily.

**Response:** More information is now added to Figures S1 and S2 captions.

**Comment 2.18**

Line 373. I am not entirely sure which overdispersion scenario the authors are referring to here. Earlier in the manuscript, two overdispersion scenarios were mentioned, could this be clarified?

**Response:** The Method and Result sections have been revised to clarify each overdispersion scenario. Here, “overdispersion” refers to how tick counts are distributed across birds (in a highly overdispersed situation, a small number of birds would be infested with a large number of ticks, while infestation would be mild for most birds). We explored two overdispersion scenarios using negative binomial distributions with overdispersion parameter values, and one no overdispersion scenario using a Poisson distribution.

Lines 382 to 390

**Comment 2.19**

Figure 1. The figure is not fully clear to me in its current form. In particular, I do not see where sample size is analysed within the figure, and it is unclear why the Poisson scenario appears only in panel C. Shouldn’t the Poisson assumption influence the estimation of other parameters as well?

**Response:** Sample size is defined as the number of birds sampled in our analysis. This is because, in most field studies that sample birds and their ticks, birds are sampled first and ticks are collected from these birds. Given that the number of ticks per birds follows a particular distribution (e.g. a negative binomial distribution, as observed in our data), the total number of birds and ticks would depend primarily on the number of birds sampled. Therefore, we treated the number of hosts sampled as the primary design target.

Lines 356 to 365

This is why we assessed the impact on the 95%HDI width and bias by the number of birds sampled on the x-axis, with the number of ticks varying accordingly as we simulated it for each sampled bird. The Poisson scenarios (red and blue squares) appear in other panels too. Their effects on other parameters were not pronounced, as reported in the main text.

Lines 469 to 479

**Comment 2.20**

Line 413. Do the authors have any suggestions regarding a possible biological explanation for this result? Even a brief hypothesis would help contextualise the finding.

**Response:** Epidemiologically, the estimated posterior probability of a *T. merula* bird being infectious for *A. phagocytophilum* essentially represents the prevalence of *A. phagocytophilum* in this species. Therefore, we believe that the similarity between our estimate and the proportion of the *T. merula* birds that tested positive for this pathogen (sampled in the same region and during similar periods, but not used for model fitting) demonstrates the robustness of our model estimates.

**Comment 2.21**

Figures 3 and 4. It would be helpful to guide the reader more explicitly in interpreting these figures. The captions describe what is shown, but provide little indication of what the patterns mean or how they should be interpreted. Adding interpretative guidance would greatly aid the reader’s understanding.

**Response:** Additional guidance has been added to the figure captions.

Lines 556, 567, 579

**Comment 2.22**

The discussion is overall complete and very well written; I only have a few minor points to raise:

Line 470. It would be helpful if the authors could more clearly describe the datasets used. For a biologist interested in applying a similar framework, having a clearer idea of the minimum data requirements would be extremely valuable. Given the strength of the work, I would also suggest briefly outlining in which situations this framework can be applied, and any limitations, to encourage cross-disciplinary uptake of the framework.

**Response:** We appreciate the reviewer’s comment. We have now provided the minimum data requirement at the beginning of the Method section, with Table 1 listing all input data and parameters used and estimated in this study. In addition, throughout the manuscript, we highlight that the framework can be applied to other host-tick systems based on data collected in field studies that sample hosts and their ticks.

Lines 177 to 182, 614 to 624

**Comment 2.23**

Line 478. As above, further clarification of the data requirements and potential constraints would strengthen the practical relevance of the work.

**Response:** Please see our response to Comment 2.22 above.

**Comment 2.24**

Line 540. The choice of using only 10 ticks, and the implications of this choice, remains not entirely clear to me. A short explanation would be useful.

**Response:** Please see our response to Comment 2.13 above.

**Comment 2.25**

Line 597. It may be worth acknowledging somewhere that studies of this type require substantial time, effort, and resources

**Response:** We have revised the manuscript to highlight these challenges.

Lines 763 to 766, 623 to 24

Reviewer #3

**Comment 3.1**

The authors present an interesting modelling framework to investigate the transmission dynamics of tick-borne pathogens and apply it to field data to estimate several epidemiologically relevant parameters. Overall, the study is of interest and the manuscript is clearly written, although several points require further clarification or detail.

**Response:** We appreciate the reviewer’s constructive and encouraging comments and suggestions. Our responses to each point are provided below. Line numbers are for the version with track changes.

**Comment 3.2**

I suggest including a schematic representation of the model structure to help readers grasp its components, as well as providing the full set of governing equations.

**Response:** Table 1 is newly included in the revised main text to show the full list of input data and the parameters estimated. Each of these parameters is directed to the corresponding equations to help readers understand the model structure.

Lines 177 to 182

**Comment 3.3**

It appears that demographic processes (such as tick and host fecundity and survival) are not included; could you clarify the rationale for omitting them? I also find it unclear why some baseline infection probabilities (e.g., α_pB, pTinf) are assumed rather than being generated by explicit infection processes. This may relate to lines 175–179, which would benefit from clearer explanation.

**Response:** Our modelling framework is designed to infer transmission probabilities at the host-tick interface using cross-sectional data collected at a sampling time point, including tick infection status and ecological covariates. Specifically, the model seeks to attribute a sampled tick’s infection to (i) transmission from its host during feeding, (ii) transmission from other ticks sampled on the same host, or (iii) infection acquired prior to the observed feeding event.

The probability that a tick is infected prior to feeding (denoted P_T,inf_ in the original manuscript, and P_L_ and P_N_ in the revised manuscript) is therefore intended to capture pathogen burden at the tick population level. We treat this probability as a baseline component in this cross-sectional framework and it is estimated from the data instead of being generated via explicit demographic and epidemiological dynamics. The intercepts are designed to capture the force of infection not captured by the bird- or tick-level coefficients.

Incorporating host and tick demographic processes (e.g. fecundity, survival, and seasonality) would require a fully dynamic transmission model and longitudinal data to inform a large number of additional parameter. At minimum, the same hosts would need to be sampled repeatedly to infer tick-to-bird transmission probability (the part noted by the reviewer regarding previous lines 175-179) or to account for the effect of ticks detached before sampling (the part as one of the limitations). However, such data are extremely challenging to collect, and without them, these processes would be weakly identifiable and therefore unlikely to provide meaningful results.

We fully agree that explicitly modelling these processes over time would be an important next step for a more complete understanding of tick-borne pathogen dynamics. However, this is beyond the scope of the present study given the available data. However, because our framework is applicable to data collected in field studies targeting specific host-tick systems, we view its potential as a complementary component that can support future work aiming to integrate longitudinal epidemiological and demographic processes into transmission models. As the first step, we have published a tick population dynamics model (Kim et al. 2025), and the present study and the findings from future research could inform how to integrate pathogen dynamics components into tick population dynamics models.

Lines 609 to 624

Kim Y, Jaulhac B, Vesga JF, Zilliox L, Boulanger N, Edmunds WJ, Métras R. Environmental drivers of Ixodes ricinus tick population dynamics: Mechanistic modelling using longitudinal field surveys and climate data. Epidemics. 2025 Dec;53:100854. doi: 10.1016/j.epidem.2025.100854. Epub 2025 Sep 8. PMID: 40945389.

**Comment 3.4**

The model includes only one host category, namely birds. As far as I know, birds are generally not the most competent reservoirs for these pathogens compared with rodents or other mammals. Could you elaborate on the justification and implications of this choice?

**Response:** Although not completely exclusive, many Borrelia genospecies show strong host associations. In Europe, *B. garinii* and *B. valaisiana* are primarily associated with birds, whereas *B. afzelii* is most commonly associated with rodents (Kurtenbach K et al. 2002). These host associations justify our focus on *B. garinii* and *B. valaisiana* in the selected bird species. In addition, our modelling framework targets “snapshot” transmission dynamics at the sampled host-tick interface, that is, transmission pathways involving the sampled host and its ticks, while allowing for the possibility that ticks may have acquired infection at earlier life stages as a baseline component in this cross-sectional framework. Therefore, the potential involvement of other host species across the tick life cycle has limited implications for our study findings. However, the framework is readily applicable to other host-tick systems, for example, to study *B. afzelii* in rodent-tick systems. The manuscript has been revised to elaborate these aspects.

Lines 614 to 620

Kurtenbach K, De Michelis S, Sewell HS, Etti S, Schäfer SM, Holmes E, Hails R, Collares-Pereira M, Santos-Reis M, Hanincová K, Labuda M, Bormane A, Donaghy M. The key roles of selection and migration in the ecology of Lyme borreliosis. Int J Med Microbiol. 2002 Jun;291 Suppl 33:152-4. doi: 10.1016/s1438-4221(02)80029-7. PMID: 12141740.

**Comment 3.5**

Equations 9 and 10 also require further clarification. FOI1 is described as the probability that birds become infected via cofeeding and/or transmission; what then is FOI2?

**Response:** ${FOI}_{2}\left[ i,k \right]$ represents the probability that tick *i* on bird *k* becomes infected through either bird-to-tick transmission, cofeeding transmission, or both, accounting for potential non-independence between the two transmission pathways and heterogeneity in transmissibility at the bird level with $\rho$ and $\eta\left[ k \right]$, while ${FOI}_{1}\left[ i,k \right]$ does not account for these factors. The manuscript has been revised to clarify these equations.

Lines 278 to 286

**Comment 3.6**

Lines 345–361 need additional methodological detail. Please provide the explicit likelihood formula and the complete list of free parameters that are estimated. What initial conditions were used? What was the simulated time span? How many MCMC iterations were run, and how was convergence assessed? I also suggest moving Figure S3 into the main text. Its caption should likely read “Model fit”.

**Response:** Additional methodological details are now provided in the revised manuscript. Input data, estimated parameters, and their definitions and priors are provided in a new Table (Table 1). More details about model fitting are also provided in the method section, with the log-likelihood function in Eq. 14. Following the reviewer suggestion, we have now moved Figure S3 into the main text.

Minor points

**Comment 3.7**

Please provide some additional details on Borrelia and Anaplasma in the introduction upon their first mention, such as their public health burden (possibly with some numbers of cases), range of competent hosts and vectors.

**Response:** Additional details on the Borrelia genospecies and Anaplasma species included in this study are now provided in the revised main text. We have also added justifications for their selection within the bird-tick system, including their specific host associations with birds, and contrasts between Borrelia and Anaplasma, and the predominance of *Ixodes ricinus* in Europe.

Lines 128 to 150

**Comment 3.8**

When were birds sampled?

**Response:** The birds and their ticks were sampled in June and July, 2023. This detail has now been provided to the manuscript.

Lines 396 to 398

**Comment 3.9**

Please explain what HDI stands for upon its first appearance.

**Response:** The full name of HDI is in the Method section. However, we have now included it in the Result section also for clarification.

**Comment 3.10**

I’d have expected some plots showing estimated probabilities as functions. Please clarify what you mean by characteristics for vectors β (eq.1, 3, 8) and list the full coefficients lists.

**Response:** We now provide the variables associated with each coefficient both in the main text and in Table 1. Please note that the estimated coefficients are provided in Figure S3 (expressed in odds ratios) and Figure S4 (expressed as raw values).

Lines 177 to 182

**Comment 3.11**

Are they free parameters to be estimate via MCMC? If so, this connects directly to the main methodological questions above.

**Response:** The full list of estimated free parameters is provided in Table 1, along with the input data.

Lines 177 to 182

**Comment 3.12**

Please note that in the provided R code there is a “setwd” on line 185 which I believe ought to be removed.

**Response:** We thank the reviewer for identifying this mistake. It has now been removed from the Supplementary code.
